# Supplementary material for: Elovl2-Ablation Leads to Mitochondrial Membrane Fatty Acid Remodeling and Reduced Efficiency in Mouse Liver Mitochondria
Source: Nutrients. 2022 Jan 27;14(3):559. doi: 10.3390/nu14030559 (PMC8838343; doi:10.3390/nu14030559)
Supplement: Supplementary file 1 [file nutrients-14-00559-s001.zip › nutrients-1533638-supplementary.pdf]

A. WT, TEM liver

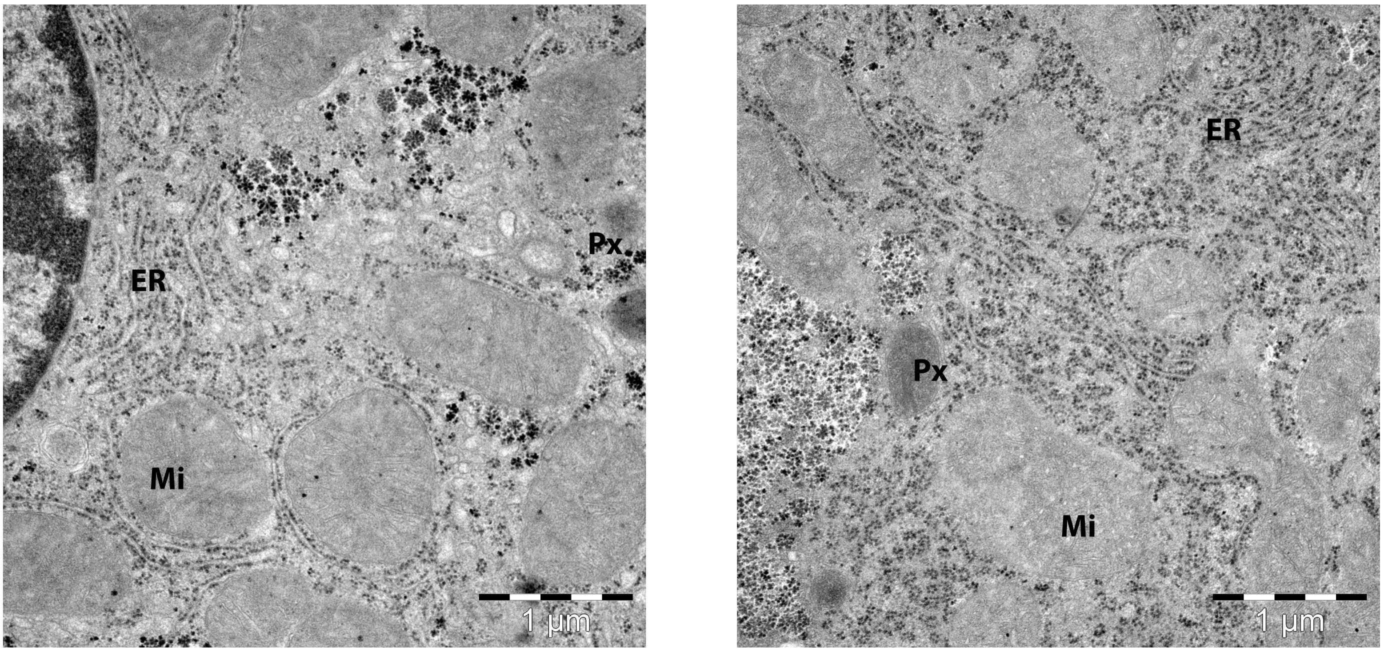

B. Elovl2 KO, TEM liver

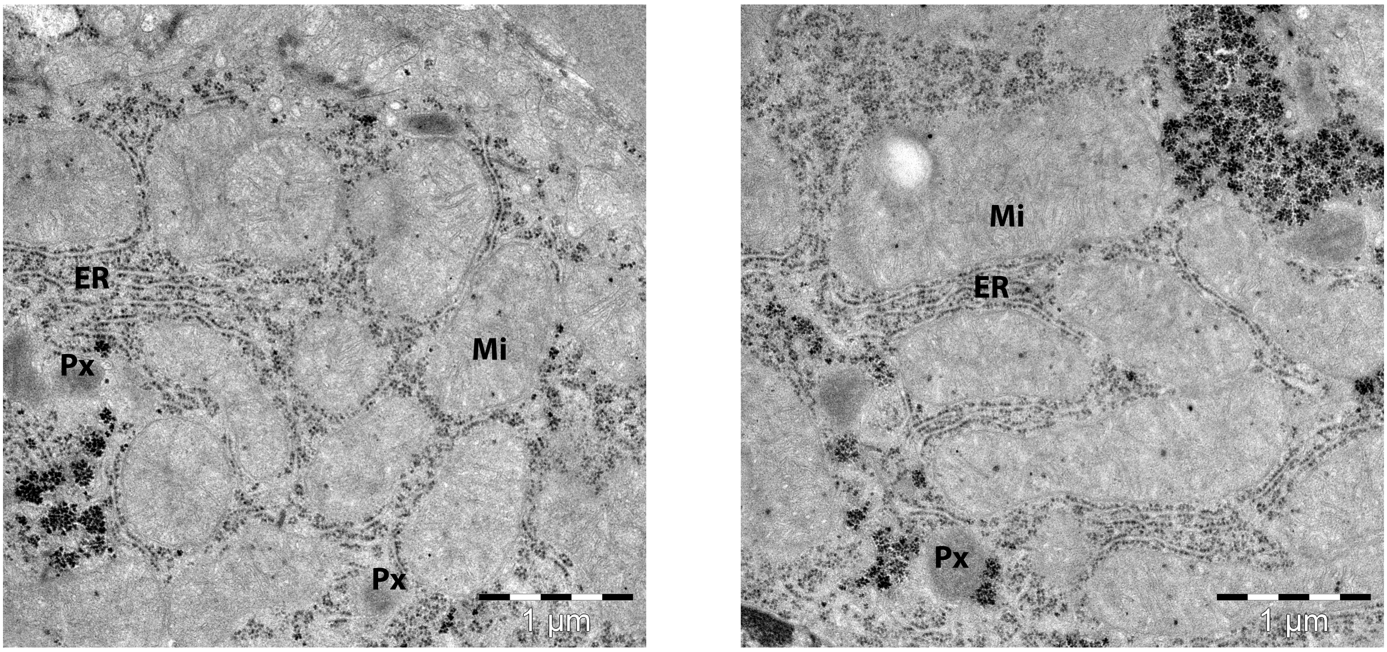

Supplementary Figure S1 Morphology of liver tissue by electron microscopy
.
(A, B). Transmission electron microscopy images of the liver of two wild-type (WT) (A) and two Elov2 KO (B) mice, showing higher number of mitochondria (Mi) and peroxisomes (Px). and no diffrence in Endoplasmic Reticulum (ER).

Table S1. Fatty acid composition in isolated mitochondria from liver of WT and Elovl2 KO mice. Graphic presentation of thse results is on Figure 2A.

Fatty acid	Genotype	N	Mean	SD	SEM	P
f14:0	WT	6	0.2680	0.0721	0.0294	0.330
	ELovl2KO	6	0.3177	0.0947	0.0387	
f16:0	WT	6	23.6732	2.5378	1.0361	0.066
	ELovl2KO	6	21.3100	1.2019	0.4907	
f16:1n-7	WT	6	1.2829	0.3468	0.1416	0.207
	ELovl2KO	6	0.9836	0.4191	0.1711	
f18:0	WT	6	11.4885	0.5138	0.2098	0.116
	ELovl2KO	6	12.4669	1.2952	0.5288	
f18:1n-9	WT	6	23.7935	4.0125	1.6381	0.001
	ELovl2KO	6	32.7314	2.5632	1.0464	
f18:2n-6	WT	6	9.7808	2.3679	0.9667	0.041
	ELovl2KO	6	7.2292	1.2288	0.5017	
f18:3n-3	WT	6	0.5593	0.1576	0.0643	0.212
	ELovl2KO	6	0.7352	0.2819	0.1151	
f18:4n-3	WT	6	0.9894	0.5604	0.2288	0.235
	ELovl2KO	6	1.2950	0.1911	0.0780	
f20:0	WT	6	0.6237	0.2909	0.1188	0.720
	ELovl2KO	6	0.6849	0.2844	0.1161	
f20:1n-9	WT	6	0.9832	0.3188	0.1302	0.608
	ELovl2KO	6	1.0666	0.2166	0.0884	
f20:2n-6	WT	6	0.5472	0.1015	0.0414	0.027
	ELovl2KO	6	0.6856	0.0823	0.0336	
f20:3n-6	WT	6	3.0741	0.6068	0.2477	0.060
	ELovl2KO	6	3.6384	0.2422	0.0989	
f20:4n-6	WTI	6	12.2402	1.0644	0.4345	0.019
	ELovl2KO	6	9.9792	1.6809	0.6863	
f20:5n-3	WT	6	0.4399	0.1272	0.0519	0.001
	ELovl2KO	6	0.7688	0.1160	0.0474	
f22:0	WT	6	0.4821	0.1907	0.0778	0.007
	ELovl2KO	6	0.8174	0.1550	0.0633	
f22:4n-6	WT	6	0.4793	0.0494	0.0202	0.077
	ELovl2KO	6	0.6258	0.1754	0.0716	
f22:5n-6	WT	6	0.8634	0.2754	0.1124	0.030
	ELovl2KO	6	0.5653	0.0853	0.0348	
f22:5n-3	WT	6	1.1844	0.3819	0.1559	0.001
	ELovl2KO	6	2.4261	0.4595	0.1876	
f24:0	WT	6	0.4104	0.0674	0.0275	0.945
	ELovl2KO	6	0.4066	0.1142	0.0466	
f22:6n-3	WT	6	6.2815	0.5643	0.2304	0.001
	ELovl2KO	6	0.7274	0.1783	0.0728	
f24:5n-3	WT	6	0.2824	0.1520	0.0621	0.942
	ELovl2KO	6	0.2756	0.1661	0.0678	
f24:6n-3	WT	6	0.2724	0.1592	0.0650	0.910
	ELovl2KO	6	0.2633	0.1081	0.0441	

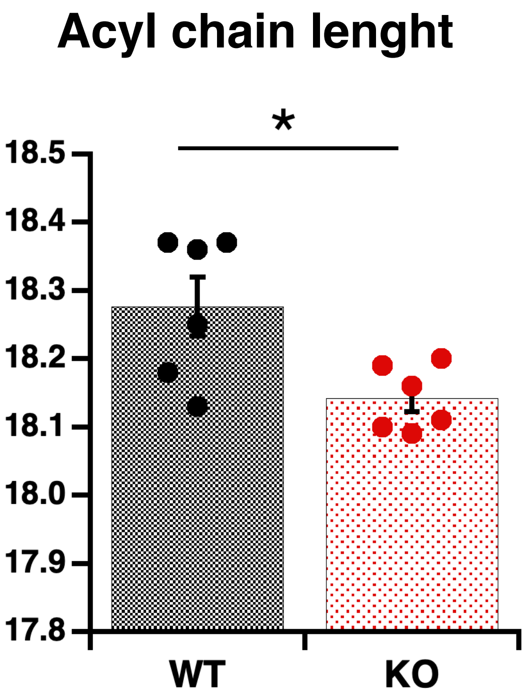

Supplementary Figure S2

Acyl chain length (ACL) of mitochondrial fatty acids from wild-type and Elovl2 KO mice.
For calculation of ACL value (number of carbon atoms), see the Materials and Methods section.
Values are means ± SEM from 6 different mitochondrial preparation for each mouse genotype.
Significant differences are shown between wild-type and Elovl2 KO mice: * $P < 0.05$.

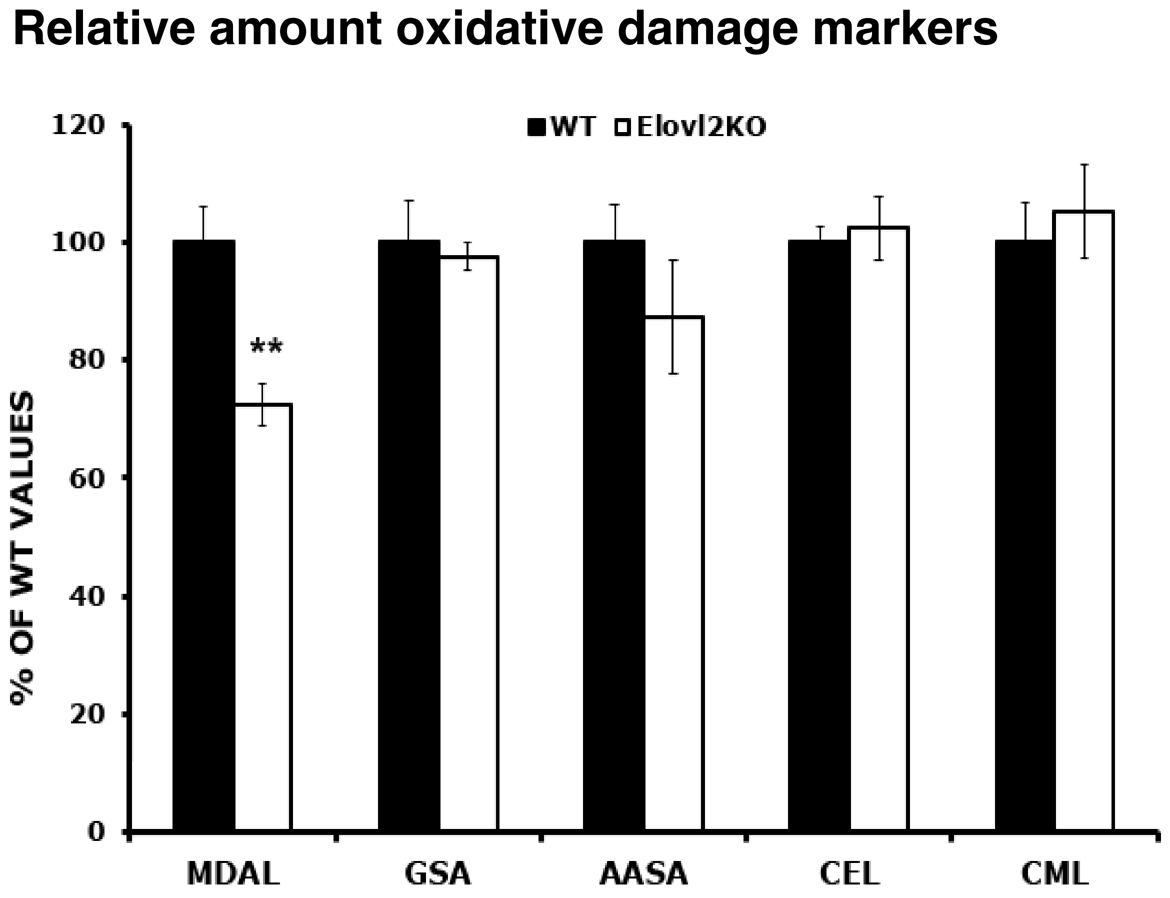

Supplementary Figure S3

Lipoxidation (MDAL), protein oxidation (GSA, AASA), and glycoxidation (CEL, CML) damage indicators in liver mitochondria from WT and Elovl2 KO mice.
Values are means ± SEM from 6 different mitochondrial preparation for each mouse genotype and expressed as a percentage of wild-type animals. Absolute values are presented in Figure 3. Significant differences are shown between wild-type and Elovl2 KO mice, ** $P < 0.01$.

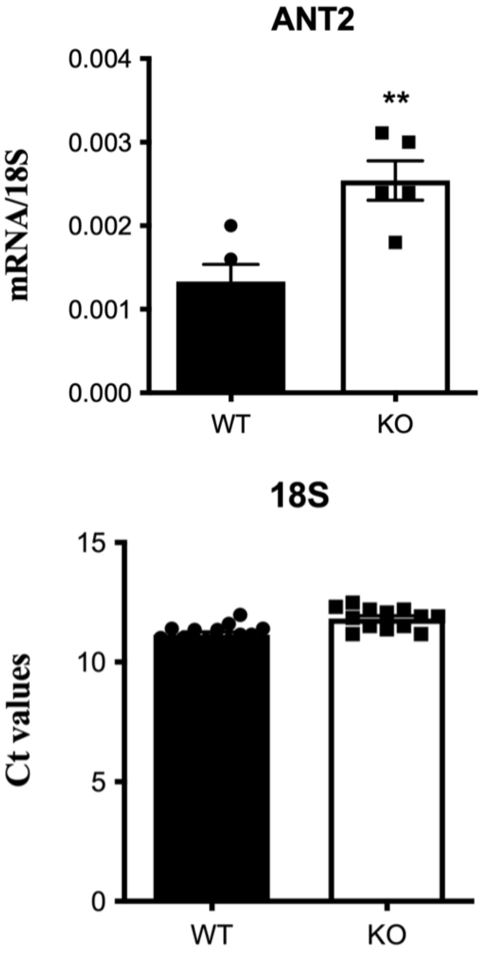

Supplementary Figure S4

Gene expression of ANT2 from WT and Elovl2 KO liver.
18S is used as house-keeping gene and all CT values are presented in the figure.
Results are shown as ± SEM from 5 different liver samples of each genotype.
Significant differences are shown between WT and Elovl2 KO mice. ** $P < 0.01$.
